# One year after the COVID-19 outbreak in Germany: long-term changes in depression, anxiety, loneliness, distress and life satisfaction

**DOI:** 10.1007/s00406-022-01400-0

**Published:** 2022-03-29

**Authors:** Christoph Benke, Lara K. Autenrieth, Eva Asselmann, Christiane A. Pané-Farré

**Affiliations:** 1 Department of Psychology, Clinical Psychology and Psychotherapy, Philipps University of Marburg, Gutenbergstraße 18, 35032 Marburg, Germany; 2 Differential and Personality Psychology, HMU Health and Medical University, Olympischer Weg 1, 14471 Potsdam, Germany; 3 Center for Mind, Brain and Behavior (CMBB), University of Marburg and Justus Liebig University, Giessen, Germany

**Keywords:** Mental health, Lockdown measures, Social distancing, Pandemic, Risk group, Longitudinal

## Abstract

Several studies have linked the COVID-19 pandemic to unfavorable mental health outcomes. However, we know little about long-term changes in mental health due to the pandemic so far. Here, we used longitudinal data from a general population sample of 1388 adults from Germany, who were initially assessed between April and May 2020 (i.e., at the beginning of the COVID-19 pandemic in Germany) and prospectively followed up after 6 (*n* = 1082) and 12 months (*n* = 945). Depressive and anxiety symptoms as well as loneliness did not change from baseline to 6-month follow-up. While anxiety symptoms did not change in the long run, depressive symptoms and loneliness increased and life satisfaction decreased from baseline to 12-month follow-up. Moreover, vulnerable groups such as younger individuals or those with a history of mental disorders exhibited an overall higher level of psychopathological symptoms across all assessment waves. Our findings suggest a deterioration in mental health during the course of the COVID-19 pandemic, which emphasizes the importance to implement targeted health promotions to prevent a further symptom escalation especially in vulnerable groups.

## Introduction

The COVID-19 pandemic and related lockdown measures have disrupted people’s everyday life. Specifically, social distancing measures have reduced social and physical activities and, thus, increased the risk of social isolation [1–3]. Moreover, financial and job insecurities as well as worries about people’s own health and the health of loved ones might have led to increased distress [4, 5]. Thus, the present pandemic situation is assumed to threaten mental health [6] especially in vulnerable populations [7, 8]. In fact, several studies reported a worldwide increase of depressive and anxiety symptoms, loneliness, and distress from the time before the pandemic to the first wave of the pandemic [see 9 for a review, 10–12]. Moreover, these longitudinal and additional cross-sectional studies have identified sociodemographic correlates and risk factors (e.g., younger age, living without a partner, a previous mental illness, lower educational level, being unemployed) of elevated distress and psychopathological symptoms during the pandemic [13–19]. These studies have helped to identify individuals at risk for short-term mental health problems at the beginning of the pandemic. However, for an adequate implementation of further lockdown measures and targeted mental health interventions, it is crucial to (a) examine long-term mental health trajectories beyond the first months of the pandemic and to (b) identify vulnerable groups with particularly unfavorable trajectories.

Recent prospective longitudinal studies investigating the course of mental health during the first months of the pandemic have demonstrated that mental health problems (i.e., general mental health and distress, as well as depressive and anxiety symptoms) decreased while lockdown measures were eased after the first COVID-19 outbreak [10, 13, 20–23]. Interestingly, the recovery of mental health problems was observed to be stronger in vulnerable populations such as women (vs. men), younger (vs. older) individuals, individuals with a lower (vs. a higher) educational level, and those with (vs. without) children [13, 22]; although the level of mental health problems remained elevated in these specific populations even after easing of the first lockdown in the UK [22]. However, after the easing of the COVID-19 situation in summer 2020, in several countries including Germany lockdown measures were repeatedly tightened and extended in response to recurrent increases in COVID-19 cases. According to vulnerability–stress models [24], one would assume that repeated distress and social isolation resulting from repeated implementation of lockdown restrictions might be associated with a worsening of mental health in the long run, especially in vulnerable groups.

However, we know little about long-term changes in mental health up to 1 year after the COVID-19 outbreak so far. Thus, it remains unresolved whether repeated implementations of lockdown restrictions do confer an increased risk for an escalation of mental health impairments in the general population and in particularly vulnerable subgroups. An improved knowledge hereon would help to inform policymakers and the health care system to implement targeted strategies to prevent adverse long-term effects on mental health.

In the current study, we analyzed data from a general population sample of 1388 adults, who were initially assessed from April to May 2020 (i.e., during the first COVID-19 wave in Germany) and prospectively followed up after 6 (i.e., from November to December 2020, during the second COVID-19 wave in Germany) and 12 months (i.e., from May to June 2021, during the third COVID-19 wave in Germany). The aim was (a) to model long-term changes in mental health up to 1 year after the initial COVID-19 outbreak in Germany and (b) to assess whether these changes were more unfavorable in particularly vulnerable groups (e.g., women, younger individuals, individuals with a previous mental illness).

## Methods

### Participants

**Fig. 1:**
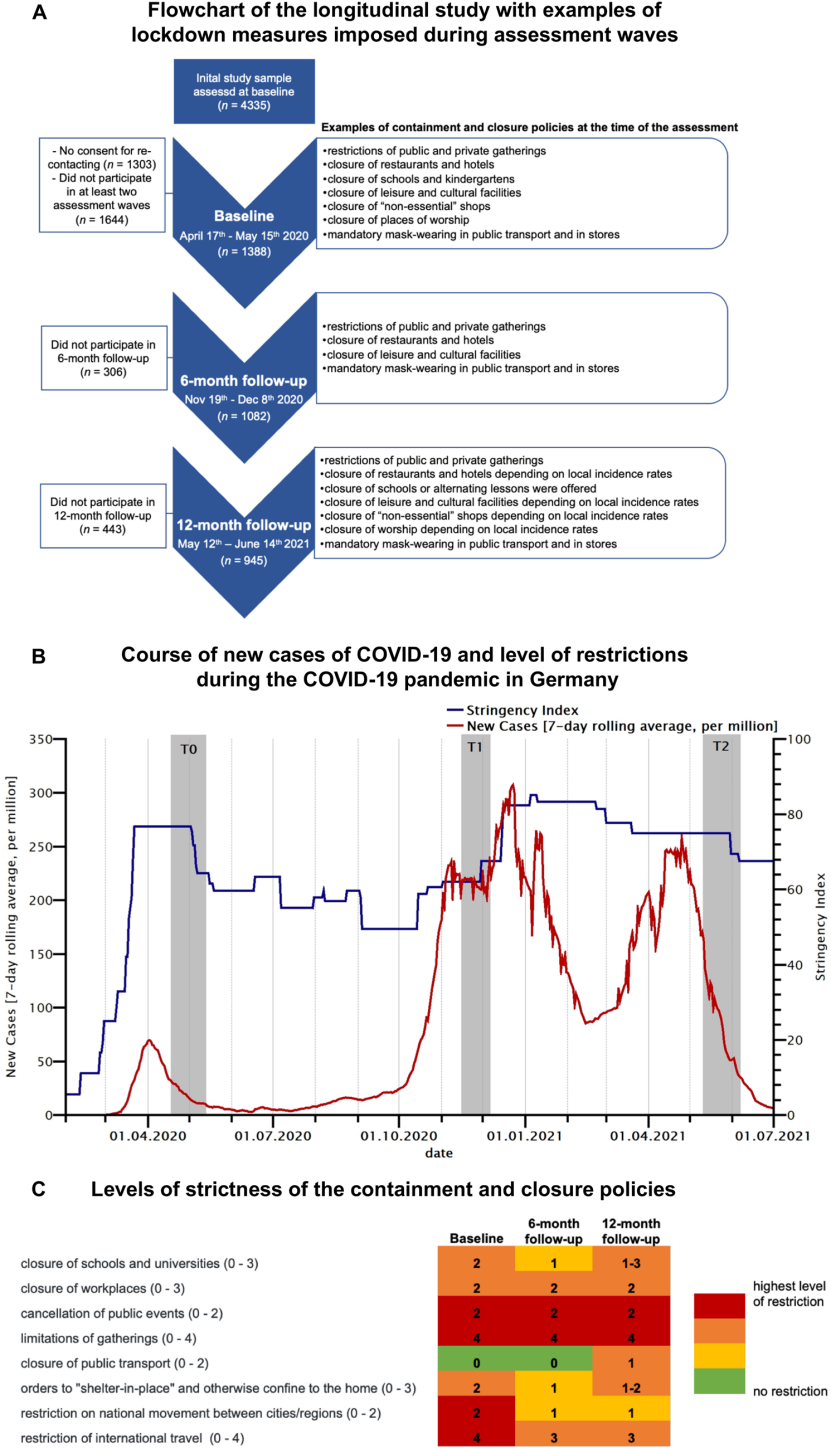
**A** Flowchart of the design of the longitudinal study with information on the study sample and examples of lockdown measures imposed during assessment time points (source: Response Measures Database (RMD) of the European Centre for Disease Prevention and Control (ECDC) and the Joint Research Centre (JRC) of the European Commission); **B** course of new cases of COVID-19 (7-day rolling average, per million) and level of restrictions due to lockdown measures (indicated by the stringency index; Oxford COVID-19 Government Response Tracker [25]) during the COVID-19 pandemic in Germany (March 2020 to July 2021). The gray bars represent the time points and durations of the three assessment waves (T0: baseline, T1: 6-month follow-up, T2: 12-month follow-up); **C** strictness of the containment and closure policies during the assessment time points (a higher score represent a higher level of strictness).The levels of strictness of the listed containment and closure policies are used to calculate the stringency index (i.e., the overall level of the government’s response, see panel B). Values in parentheses represent the range of the restriction due to the containment and closure policies. Please see [25] for further information on the coding of the different levels of strictness. Data were obtained from the Oxford COVID-19 Government Response Tracker [25]

In the present study, we used data from a non-probability sample of the general population in Germany assessed at the beginning of the pandemic (see [14]) and then prospectively followed up after 6 and 12 months. In the present study, we only consider data from those individuals who participated in at least two assessment time points. In this longitudinal study, a total of 1388 participants repeatedly completed an online survey (soscisurvey.de) over 1 year (see panel A of Fig. 1 for an overview of the study design and study sample). The first assessment (baseline; *n* = 1388) started during the first peak of the COVID-19 pandemic in Germany, between April 17th and May 15th 2020, that is, four weeks after all German federal states had implemented public health measures (see Fig. 1 for further information on the containment measures imposed at the time of the assessment). The second assessment (6-month follow-up; *n* = 1082) was conducted between November 19th and December 8th 2020. At this time, COVID-19 cases rapidly increased and lockdown measures were extended and tightened (see Fig. 1). The third assessment (1-year follow-up, *n* = 945) was conducted between May 12th and June 14th 2021, that is, in the end of the third COVID-19 wave in Germany (see Fig. 1). As can be seen in Fig. 1B and C, the severity of lockdown-related restrictions during the 1-year follow-up was comparable to the severity of lockdown-related restrictions during baseline. However, at the 6-month follow-up, the overall stringency index and level of strictness of certain containment measures were slightly lower relative to the time of the baseline and 12-month follow-up assessment (see Fig. 1B and C). A total of 639 individuals participated at all three assessment time points. Participants were recruited via convenience sampling methods (e.g., via social media, personal contacts, or email). All participants provided informed consent. The study was approved by the local Ethics Committee of the University of Marburg (2020-33k**)**.

### Measures

At baseline, several sociodemographic factors were assessed (see Table 1). We also asked participants to indicate whether they do or do not belong to an officially designated risk group for a severe COVID-19 disease progression (COVID-19 risk group). Moreover, the following psychological outcomes were measured:

**Table 1:** Associations of assessment time, sociodemographic and COVID-19-related predictors with psychological outcome measures

Coefficients	*n* (%)	Depression	Anxiety	Loneliness	Psychosocial distress	Life satisfaction
		b (SE)	b (SE)	b (SE)	b (SE)	b (SE)
Wave						
T0 (reference)	1388					
T1	1082	0.15 (0.13)	−0.04 (0.11)	– 0.09 (0.05)	-0.49 (0.10)***	-0.50 (0.06)***
T2	945	0.65 (0.13)***	0.19 (0.12)	0.16 (0.05)**	– 0.29 (0.10)**	– 0.67 (0.07)***
Gender						
Male (reference)	272 (19.6%)					
Female	1116 (80.4%)	0.44 (0.29)	0.31 (0.26)	– 0.03 (0.11)	0.85 (0.22)***	0.28 (0.12)*
Age						
18–34 (reference)	449 (32.3%)					
35–49	546 (39.3%)	– 0.98 (0.30)**	– 0.59 (0.26)*	– 0.27 (0.12)*	0.27 (0.22)	0.01 (0.13)
50–64	347 (25.0%)	– 2.29 (0.34)***	– 1.78 (0.29)***	– 0.47 (0.13)***	– 0.41 (0.25)	0.46 (0.14)**
65 +	46 (3.3%)	– 4.15 (0.72)***	– 3.30 (0.63)***	– 0.41 (0.28)	– 1.92 (0.53)***	1.13 (0.30)***
Educational level						
Low (reference)	36 (2.6%)					
Middle	696 (50.1%)	– 1.77 (0.75)*	– 1.55 (0.65)*	– 0.38 (0.29)	– 0.89 (0.55)	1.06 (0.31)**
High	656 (47.3%)	– 2.68 (0.75)***	– 2.00 (0.65)**	– 0.44 (0.29)	– 1.44 (0.55)**	1.29 (0.32)***
Employment						
Employed (reference)	1145 (82.5%)					
Unemployed/non-working	243 (17.5%)	0.29 (0.33)	0.55 (0.29)	0.19 (0.13)	– 0.10 (0.24)	– 0.23 (0.14)
Relationship						
Single (reference)	459 (33.1%)					
Partnership—living together	121 (8.7%)	– 0.56 (0.33)	– 0.05 (0.29)	– 0.16 (0.13)	0.25 (0.24)	0.48 (0.14)**
Partnership—not living together	808 (58.2%)	– 0.71 (0.44)	– 0.06 (0.38)	– 0.15 (0.17)	– 0.17 (0.32)	0.25 (0.18)
Living alone						
No	1053 (75.9%)					
Yes	335 (24.1%)	0.43 (0.36)	0.14 (0.31)	0.33 (0.14)*	0.07 (0.27)	– 0.35 (0.15)*
Living with underage children						
No	931 (67.1%)					
Yes	457 (32.9%)	– 0.06 (0.29)	0.28 (0.25)	0.14 (0.11)	1.03 (0.21)***	0.13 (0.12)
Current or previous psychiatric/psychotherapeutic treatment						
No	817 (58.9%)					
Previous	343 (24.7%)	2.12 (0.28)***	1.94 (0.24)***	0.29 (0.11)**	1.39 (0.21)***	– 0.49 (0.12)***
Current	227 (16.5%)	4.81 (0.33)***	3.93 (0.29)***	0.67 (0.13)***	2.89 (0.25)***	– 1.16 (0.14)***
COVID-19 risk group – self-reported						
No	861 (62.0%)					
Yes	527 (38.0%)	0.98 (0.27)**	0.66 (0.23)**	– 0.02 (0.10)	0.86 (0.20)***	– 0.011 (0.11)

Values in bold type indicate statistical significance (*p* < 0.05)

b: Estimated fixed effects; ***p < 0.001, **p < 0.01, *p < 0.05

Depressive symptoms were assessed with the Patient Health Questionnaire-9 (PHQ-9; [26]). Generalized anxiety was assessed with the 7-item Generalized Anxiety Disorder scale (GAD-7; [27, 28]). Loneliness was assessed with the 3-item version of the UCLA Loneliness Scale [29]. Psychosocial distress (e.g., due to financial problems or worries, distress at work, distress resulting from childcare) was assessed with the Stress module of the Patient Health Questionnaire. Finally, and as in previous research (see [30]), general life satisfaction was assessed with a single item (“All things considered, how satisfied are you with your life these days?”) and a 11-point Likert-scale ranging from 0 (completely dissatisfied) to 10 (completely satisfied).

### Data analysis

Statistical analyses were conducted with SPSS 26 (SPSS for Windows, IBM). All analyses were conducted using mixed regression models with repeated measurement occasions (i.e., assessment time points, Level 1) nested or clustered within persons (Level 2). In all analyses, fixed-effect regression models with an underlying compound symmetry covariance matrix and a restricted maximum likelihood estimation were used. To examine the change of psychological outcomes from the baseline to the 6-month and 12-month follow-up assessment, the assessment time point was dummy-coded (T0 vs. T1 and T0 vs. T2) and both dummy-coded variables were entered as continuous predictors into the regression models. First, psychological outcome measures were regressed on sociodemographic/risk factors and the dummy-coded assessment time point (T0 vs. T1 and T0 vs. T2) as multiple predictors. Second, interaction terms between the dummy-coded assessment time point and each sociodemographic/risk factor were computed and added to the analyses to explore whether symptom changes differed between individuals with and without specific risk factors. The alpha level was set at 0.05. Our main analyses on the effects of sociodemographic factors and the assessment time point on mental health refer to nine different sociodemographic factors (gender, age, educational level, employment, relationship, living alone, living with underage children, current or previous psychiatric/psychotherapeutic treatment, COVID-19 risk group) and two dummy-coded timing variables (T0 vs. T1, T0 vs. T2) * five outcomes (depressive symptoms, anxiety symptoms, loneliness, distress, and life satisfaction). Our main analyses on the interaction effects of sociodemographic factors on the change of mental health from baseline to the 6-month and 12-month follow-up refer to two time-dependent effects (6-month follow-up, 12-month follow-up) * five outcomes (depressive symptoms, anxiety symptoms, loneliness, distress, and life satisfaction) * nine different sociodemographic factors (gender, age, educational level, employment, relationship, living alone, living with underage children, current or previous psychiatric/psychotherapeutic treatment, COVID-19 risk group). We did not adjust for multiple testing because each effect refers to another research question based on clearly distinguishable constructs [31]. However, researchers who believe that adjustment for multiple testing is necessary may refer to this number of effects.

## Results

### Effect of sociodemographic/risk factors on psychological outcomes

Associations between sociodemographic/risk factors and psychological outcomes are presented in Table 1. Younger age, a lower educational level, a history of mental disorders and belonging to a COVID-19 risk group were associated with increased anxiety and depressive symptoms. Younger age, living alone and a history of mental disorders were associated with higher loneliness. Female sex, younger age, lower educational level, cohabiting with children, a history of mental disorders and belonging to a COVID-19 risk group were associated with elevated psychosocial distress. Female sex, older age, higher educational level, cohabiting with a partner and no history of mental health disorders were associated with higher life satisfaction.

### Longitudinal change from baseline in depressive and anxiety symptoms, loneliness, distress and life satisfaction

As shown in Table 1, depressive symptoms (*b* = 0.15, *SE* = 0.13, *p* = 0.247) and loneliness (*b* = − 0.09, *SE* = 0.05, *p* = 0.071) did not change significantly from baseline to 6-month follow-up (see also Table 2 for means and standard deviations of the respective outcomes). However, depressive symptoms (*b* = 0.65, *SE* = 0.13, *p* < 0.001) and loneliness (*b* = 0.16, *SE* = 0.05, *p* = 0.003) increased from baseline to 1-year follow-up. Psychosocial distress and life satisfaction decreased from baseline to 6-month (*ps* < 0.001) and 1-year follow-up (*ps* < 0.015). Anxiety symptoms did not change significantly over time (*p*s > 0.05, see Table 1).

**Table 2:** Means and standard deviations of depression, anxiety, loneliness, psychosocial distress and life satisfaction at the baseline (T0), 6-month (T1) and 12-month (T2) follow-up assessment in the total sample and sample subgroups

	Depression			Anxiety			Loneliness			Distress			Life satisfaction		
	T0	T1	T2	T0	T1	T2	T0	T1	T2	T0	T1	T2	T0	T1	T2
Overall	7.4 (5.3)	7.6 (5.5)	7.9 (5.4)	5.8 (4.6)	5.8 (5.5)	5.9 (5.5)	5.8 (2.0)	5.7 (1.9)	6.0 (1.9)	6.9 (3.9)	6.3 (3.8)	6.5 (3.8)	6.5 (2.2)	6.0 (2.3)	5.9 (2.3)
Gender															
Male	7.2 (5.4)	7.1 (5.6)	7.4 (5.4)	5.8 (4.9)	5.2 (4.9)	5.6 (4.6)	5.9 (2.0)	5.7 (2.0)	6.1 (1.9)	6.1 (3.7)	5.3 (3.4)	5.7 (3.7)	6.2 (2.4)	5.7 (2.6)	5.6 (2.4)
Female	7.5 (5.3)	7.7 (5.4)	8.0 (5.5)	5.8 (4.5)	5.9 (4.7)	6.0 (4.6)	5.8 (1.9)	5.7 (1.9)	5.9 (1.9)	7.1 (4.0)	6.6 (3.9)	6.7 (3.9)	6.6 (2.2)	6.1 (2.3)	6.0 (2.2)
Age															
18–34 (reference)	8.5 (5.5)	8.9 (5.9)	8.9 (5.7)	6.4 (4.7)	6.9 (5.1)	6.6 (6.6)	6.1 (1.9)	6.0 (1.8)	6.2 (1.8)	6.6 (3.8)	6.2 (3.7)	6.3 (3.8)	6.2 (2.2)	5.7 (2.3)	5.8 (2.1)
35–49	7.3 (5.0)	7.4 (5.2)	7.9 (5.2)	6.0 (4.5)	5.7 (4.7)	6.0 (4.4)	5.7 (2.0)	6.7 (2.0)	6.9 (1.9)	7.4 (3.9)	6.7 (3.9)	7.0 (3.8)	6.5 (2.2)	6.1 (2.3)	5.8 (2.2)
50–64	6.5 (5.2)	6.5 (5.0)	7.2 (5.4)	5.0 (5.3)	4.8 (4.2)	5.3 (4.4)	5.5 (2.0)	5.5 (1.9)	5.7 (2.0)	6.6 (4.2)	6.2 (3.6)	6.5 (3.9)	6.9 (2.2)	6.2 (2.4)	6.0 (2.4)
65 +	5.3 (5.4)	3.8 (3.2)	5.2 (4.2)	4.0 (4.0)	3.0 (2.8)	3.7 (3.4)	5.8 (2.0)	5.4 (2.1)	6.1 (2.0)	5.3 (4.2)	3.9 (3.6)	4.0 (3.1)	6.9 (2.6)	7.1 (2.2)	6.8 (2.3)
Educational level															
Low (reference)	10.6 (7.1)	9.6 (6.2)	8.9 (6.5)	8.3 (6.1)	7.2 (5.1)	7.4 (5.8)	6.3 (2.3)	6.4 (1.9)	6.1 (2.2)	8.0 (4.7)	7.3 (3.7)	7.8 (5.3)	5.0 (2.8)	4.6 (3.0)	5.5 (2.9)
Middle	7.9 (5.6)	8.2 (5.8)	8.7 (5.7)	6.1 (4.6)	6.2 (4.9)	6.4 (4.7)	5.9 (2.0)	5.8 (2.0)	6.0 (1.9)	7.3 (4.0)	6.7 (3.8)	6.9 (3.7)	6.3 (2.3)	5.9 (2.4)	5.7 (2.3)
High	6.7 (4.8)	6.8 (5.0)	7.0 (5.0)	5.4 (4.4)	5.3 (4.5)	5.4 (4.4)	5.7 (1.9)	5.7 (1.8)	5.9 (1.9)	6.4 (3.8)	5.9 (3.8)	6.1 (3.9)	6.8 (2.1)	6.2 (2.2)	6.1 (2.2)
Employment															
Employed (reference)	7.2 (5.1)	7.4 (5.3)	7.6 (5.2)	5.6 (4.4)	5.6 (4.7)	5.7 (4.4)	5.7 (1.9)	5.7 (1.9)	5.9 (1.9)	6.8 (3.9)	6.3 (3.8)	6.5 (3.8)	6.6 (2.1)	6.1 (2.3)	5.9 (2.2)
Unemployed/non-working	8.6 (5.9)	8.4 (6.2)	9.2 (6.4)	6.8 (5.1)	6.6 (5.2)	7.1 (5.3)	6.2 (2.1)	5.9 (2.0)	6.3 (2.0)	7.2 (4.2)	6.5 (4.0)	6.4 (4.0)	5.9 (2.5)	5.8 (2.5)	5.5 (2.6)
Relationship															
Single (reference)	8.7 (5.7)	8.6 (5.7)	8.7 (5.9)	6.4 (4.8)	6.3 (5.0)	6.2 (4.7)	6.1 (1.9)	6.0 (1.9)	6.2 (1.9)	6.8 (3.7)	6.3 (3.6)	5.4 (3.7)	5.9 (2.4)	5.5 (2.4)	5.4 (2.3)
Partnership—living together	6.7 (4.9)	7.0 (5.2)	7.5 (5.2)	5.5 (4.4)	5.5 (4.6)	5.8 (4.5)	5.6 (1.9)	5.5 (1.9)	5.8 (1.9)	6.9 (4.0)	6.5 (4.0)	6.7 (3.9)	6.9 (2.0)	6.3 (2.2)	6.2 (2.2)
Partnership—not living together	7.3 (5.4)	7.1 (5.6)	8.0 (5.4)	5.6 (4.7)	5.8 (4.8)	6.0 (4.7)	5.8 (2.0)	5.8 (2.1)	6.0 (2.1)	6.5 (4.1)	5.6 (3.3)	6.3 (3.7)	6.4 (2.4)	6.0 (2.4)	5.6 (2.5)
Living alone															
No	7.0 (5.1)	7.3 (5.3)	7.6 (5.3)	5.6 (4.5)	5.7 (4.6)	5.8 (4.5)	5.7 (1.9)	5.6 (1.9)	5.8 (1.9)	6.9 (4.0)	6.4 (3.9)	6.6 (3.9)	6.8 (2.0)	6.2 (2.2)	6.1 (2.1)
Yes	8.6 (5.8)	8.4 (6.0)	9.0 (6.0)	6.4 (4.9)	6.2 (5.1)	6.2 (4.8)	6.2 (2.1)	6.1 (1.9)	6.3 (1.9)	6.7 (3.8)	6.2 (3.4)	6.4 (3.7)	5.8 (2.5)	5.5 (2.5)	5.2 (2.4)
Living with underage children															
No	7.6 (5.4)	7.8 (5.6)	8.1 (5.6)	5.8 (4.6)	5.9 (4.9)	5.9 (4.7)	5.8 (2.0)	5.8 (1.9)	5.9 (1.9)	6.4 (3.9)	6.1 (3.7)	6.2 (3.8)	6.4 (2.3)	5.9 (2.4)	5.8 (2.3)
Yes	7.1 (5.1)	7.0 (5.1)	7.6 (5.2)	6.0 (4.5)	5.6 (4.5)	6.1 (4.4)	5.8 (2.0)	5.6 (1.9)	6.0 (1.9)	7.7 (4.0)	6.9 (4.0)	7.3 (4.0)	6.8 (2.0)	6.4 (2.2)	6.0 (2.1)
Current or previous psychiatric/psychotherapeutic treatment															
No	5.8 (4.4)	6.0 (4.6)	6.5 (4.7)	4.5 (3.8)	4.5 (4.1)	4.8 (3.8)	5.6 (1.9)	5.5 (1.9)	5.7 (1.8)	6.0 (3.6)	5.5 (3.5)	5.7 (3.6)	7.0 (1.9)	6.4 (2.2)	6.2 (2.1)
Previous	8.3 (5.4)	8.6 (5.5)	8.7 (5.5)	6.8 (4.8)	6.7 (4.9)	6.5 (4.7)	5.9 (2.0)	5.9 (1.9)	6.1 (2.0)	7.5 (4.0)	7.0 (3.9)	7.1 (3.8)	6.2 (2.3)	5.8 (2.4)	5.8 (2.4)
Current	11.7 (5.7)	11.5 (5.9)	11.7 (5.9)	9.1 (4.9)	8.8 (5.1)	9.0 (5.3)	6.4 (2.0)	6.3 (1.9)	6.6 (1.8)	9.1 (4.1)	8.5 (3.8)	8.6 (3.9)	5.2 (2.5)	5.1 (2.4)	5.0 (2.4)
COVID-19 risk group															
No	7.1 (5.0)	7.5 (5.3)	7.7 (5.5)	5.7 (4.4)	5.7 (4.8)	5.8 (4.5)	5.8 (2.0)	5.7 (1.9)	6.0 (1.9)	6.6 (3.8)	6.0 (3.8)	6.2 (3.8)	6.6 (2.1)	6.0 (2.3)	5.9 (2.2)
Yes	7.9 (5.8)	7.7 (5.4)	8.2 (5.5)	6.1 (4.8)	6.0 (4.8)	6.2 (4.8)	5.8 (1.9)	5.7 (1.9)	5.7 (1.9)	7.3 (4.1)	6.9 (3.9)	7.0 (3.9)	6.4 (2.3)	6.0 (2.4)	5.9 (2.3)

### Effects of sociodemographic and COVID-19-related factors on the change in depressive and anxiety symptoms, loneliness, distress and life satisfaction

As shown in Table 2, older individuals (aged 65 +) and those belonging to a COVID-19 risk group reported a reduction of depressive symptoms from baseline to 6-month follow-up, while younger individuals (aged 18–34 years) and those who did not belong to a COVID-19 risk group showed a slight increase of depression (*b* = − 1.47, *SE* = 0.74, *p* = 0.046 for age x assessment wave, *b* = − 0.64, *SE* = 0.26, *p* = 0.015 for COVID-19 risk group x assessment time point). Persons without a history of mental disorders experienced a strong increase of depressive symptoms from baseline to 12-month follow-up, while depressive symptomatology remained stable on a high level in individuals who received psychiatric/psychological treatment (*b* = − 1.04, *SE* = 0.37, *p* = 0.005). Men, older individuals (aged 35 years and above), and individuals with underage children experienced a decrease of anxiety symptoms from baseline to 6-month follow-up, while women, younger individuals, and individuals without underage children experienced a slight increase of anxiety (by assessment time point interactions: *p*s < 0.030; see Table 3). There was a stronger decrease in psychological distress from baseline to the 6-month follow-up in individuals with (vs. without) underage children (*b* = − 0.56, *SE* = 0.20, *p* = 0.006) as well as a stronger reduction in distress from baseline to 12-month follow-up in unemployed (vs. employed) individuals (*b* = − 0.62, *SE* = 0.27, *p* = 0.021). Employed (vs. unemployed) and those individuals without (vs. with) a history of mental disorders showed a stronger decrease in life satisfaction from baseline to the 6-month and 12-month follow-up (*p*s < 0.034, see Table 3). Life satisfaction decreased from baseline to 12-month follow-up in those with a high (vs. low) educational level (*b* = − 1.03, *SE* = 0.49, *p* = 0.036). Unemployed/non-working individuals showed a stronger reduction in loneliness from baseline to the 6-month follow-up assessment than employed individuals (*b* = − 0.35, *SE* = 0.13, *p* = 0.008). Other variables did not modulate the change in depressive and anxiety symptoms, loneliness, distress or life satisfaction (see Table 3).

**Table 3:** Change in psychological outcome measures from baseline to 6-month and 12-month follow-up between sample subgroups

Predictor	Change from baseline to 6-month follow-up					Change from baseline to 1-year follow-up				
	Depression	Anxiety	Loneliness	Psychosocial distress	Life satisfaction	Depression	Anxiety	Loneliness	Psychosocial distress	Life satisfaction
	b (SE)	b (SE)	b (SE)	b (SE)	b (SE)	b (SE)	b (SE)	b (SE)	b (SE)	b (SE)
Female (vs. male)	0.50 (0.33)	0.63 (0.29)*	0.09 (0.13)	0.25 (0.25)	– 0.09 (0.16)	0.46 (0.34)	0.58 (0.30)	0.02 (0.14)	0.21 (0.26)	– 0.08 (0.17)
Age (reference is 18–34 years)										
35–49	– 0.21 (0.30)	– 0.59 (0.27)*	0.04 (0.12)	– 0.08 (0.23)	0.07 (0.15)	0.02 (0.32)	– 0.35 (0.28)	0.09 (0.13)	0.06 (0.24)	– 0.19 (0.16)
50–64	– 0.39 (0.34)	– 0.69 (0.30)*	0.06 (0.14)	0.03 (0.26)	– 0.13 (0.17)	0.14 (0.36)	– 0.14 (0.32)	0.07 (0.14)	0.29 (0.27)	– 0.34 (0.18)
65 +	– 1.47 (0.74)*	– 1.09 (0.66)	– 0.32 (0.30)	– 0.82 (0.55)	0.66 (0.37)	– 0.87 (0.75)	– 0.78 (0.67)	0.18 (0.30)	– 0.89 (0.56)	0.42 (0.37)
Educational level (reference is low)										
Middle	1.21 (0.80)	1.14 (0.71)	– 0.23 (0.32)	0.11 (0.60)	0.08 (0.40)	1.54 (0.99)	0.72 (0.88)	0.25 (0.40)	– 0.87 (0.74)	– 0.88 (0.49)
High	0.92 (0.80)	0.88 (0.71)	– 0.16 (0.32)	0.28 (0.60)	0.01 (0.40)	1.25 (0.99)	0.60 (0.88)	0.27 (0.40)	– 0.77 (0.74)	– 1.03 (0.49)*
Unemployed/non-working (vs. employed)	– 0.52 (0.33)	– 0.15 (0.30)	– 0.35 (0.13)**	– 0.31 (0.25)	0.54 (0.17)**	0.03 (0.36)	0.17 (0.32)	– 0.04 (0.14)	– 0.62 (0.27)*	0.38 (0.18)*
Relationship (reference is single)										
Partnership – living together	0.31 (0.28)	0.09 (0.25)	– 0.00 (0.20)	0.06 (0.21)	– 0.16 (0.14)	0.40 (0.29)	0.38 (0.26)	0.09 (0.12)	0.38 (0.22)	– 0.26 (0.15)
Partnership – not living together	– 0.17 (0.49)	0.29 (0.44)	0.05 (0.20)	– 0.27 (0.37)	– 0.06 (0.24)	0.16 (0.50)	0.54 (0.45)	0.07 (0.20)	0.13 (0.38)	– 0.17 (0.25)
Living alone (vs. not living alone))	– 0.30 (0.30)	– 0.11 (0.27)	– 0.00 (0.12)	0.03 (0.22)	0.22 (0.15)	– 0.18 (0.31)	– 0.50 (0.28)	– 0.06 (0.12)	– 0.16 (0.23)	0.07 (0.16)
Living with (vs. without) underage children	– 0.46 (0.27)	– 0.54 (0.24)*	– 0.19 (0.11)	– 0.56 (20)**	0.15 (0.14)	– 0.18 (0.28)	– 0.05 (0.25)	– 0.02 (0.11)	– 0.17 (0.21)	– 0.13 (0.14)
Current or previous psychiatric/psychotherapeutic treatment (reference is no)										
Previous	– 0.13 (0.31)	– 0.30 (0.27)	0.07 (0.12)	– 0.16 (0.23)	0.19 (0.15)	– 0.26 (0.32)	– 0.51 (0.29)	0.02 (0.13)	0.01 (0.24)	0.40 (0.16)*
Current	– 0.43 (0.35)	– 0.23 (0.31)	0.01 (0.14)	– 0.15 (0.27)	0.50 (0.18)**	– 1.04 (0.37)**	– 0.63 (0.33)	– 0.03 (0.15)	– 0.43 (0.28)	0.77 (0.19)***
COVID-19 risk group (vs. no risk group)	– 0.64 (0.26)*	– 0.11 (0.23)	– 0.06 (0.10)	0.13 (0.20)	0.22 (0.13)	– 0.20 (0.28)	– 0.03 (0.25)	– 0.01 (0.11)	0.19 (0.21)	0.16 (0.14)

Values in bold type indicate statistical significance (*p* < 0.05)

b: estimated fixed interaction effects; ***p < 0.001, **p < 0.01, *p < 0.05

## Discussion

Studies investigating the long-term consequences of the ongoing COVID-19 pandemic on mental health are still rare. However, the study of potential long-term consequences of the COVID-19 pandemic is important to inform the health care system and to implement preventive strategies to reduce potential negative mental health consequences. Therefore, we investigated how depression, anxiety, distress, loneliness and life satisfaction longitudinally changed over the course of 1 year, from the first to the second and third wave of the pandemic. Moreover, we investigated whether longitudinal changes differed between individuals with vs. without specific sociodemographic characteristics and risk factors (e.g., women vs. men and younger vs. older individuals).

The present study documents a long-term deterioration of mental health during the COVID-19 pandemic in Germany. Specifically, we observed an increase of depressive symptoms and loneliness as well as a decrease in life satisfaction from the beginning of the COVID-19 pandemic to the 1-year follow-up. Anxiety symptoms persisted on a high level over the 1-year follow-up period. In contrast to these long-term effects, we found no change in loneliness, anxiety, and depressive symptoms in the short run (i.e., from baseline to the 6-month follow-up assessment), corroborating previous longitudinal data using a 6-month follow-up period [32, 33]. However, life satisfaction and psychosocial distress decreased during the same period. Moreover, we identified vulnerable groups (e.g., younger individuals) who were at increased risk for an overall higher level of psychopathological symptoms across all assessment time points but also for a short-term deterioration of mental health problems.

In the present study, depressive symptoms did not change in the short run (i.e., from the first to the second COVID-19 wave in Germany). However, after 1 year, we observed a worsening of depressive symptoms relative to the beginning of the COVID-19 pandemic in Germany which is in line with evidence from a longitudinal study among US adults [34]. Moreover, our data are in line with findings from a longitudinal population-based survey (COVID-19 Snapshot Monitoring) in Germany demonstrating that, at the time of our 12-month follow-up assessment, individuals felt more burdened than during the baseline and 6-month follow-up assessment [35, 36]. Importantly, previous longitudinal studies conducted before the COVID-19 pandemic (i.e., under non-pandemic conditions) did not observe such significant changes in mental health problems over time [13], suggesting that the increase in depressive symptoms during the COVID-19 pandemic is not the result of annual or seasonal variations. It is to note that, between the 6-month follow-up and 12-month follow-up assessment, two long-lasting and highly restrictive lockdowns were imposed in response to increases in COVID-19 cases in Germany. However, the degree of lockdown-related restrictions during the 1-year follow-up assessment was comparable to the level of restrictions being present during the baseline assessment (see Fig. 1). One might suggest that repeated and long-lasting restrictions and isolations led to an increase in depressive symptoms, while anxiety symptoms persisted on a high level over the 1-year follow-up period. Most interestingly, the increase in depression was accompanied by an increase in loneliness and a reduction in life satisfaction. In contrast, during the same period, general psychosocial distress continuously decreased. Thus, the present data might suggest that deterioration of depressive symptoms during the pandemic is rather linked to increased loneliness and lower life satisfaction in response to reduction of social contacts and social isolation but not to an overall higher level of psychosocial distress related to the pandemic situation. This finding corresponds to previous studies that demonstrated that loneliness and social isolation are important risk factors for the onset or increase in depressive symptoms [37–40]. Moreover, the worsening of depressive symptoms and loneliness in the long run was preceded by a decline in life satisfaction indicating that life satisfaction may serve as a sensitive marker or early indicator for a subsequent deterioration of psychopathological symptoms [41].

In line with evidence from several cross-sectional and longitudinal studies worldwide [13–16, 18–20, 22], we demonstrated that, across all assessment waves, being young, a lower educational level, a history of mental disorders and belonging to a COVID-19 risk group are risk factors for high levels of depression, anxiety, distress and decreased life satisfaction during the pandemic. Moreover, being young, living alone and a history of mental disorders were associated with increased loneliness. Corroborating previous position papers that predicted an increase of mental health problems in specific populations [7, 8], we identified vulnerable groups with particularly unfavorable trajectories in the short term. For example, we found that younger individuals showed an increase in depressive and anxiety symptoms while depression and anxiety symptoms decreased in older individuals. Moreover, females reported a slight increase in anxiety, while males exhibited a decrease in anxiety symptoms. These findings suggest that especially vulnerable groups fail to cope with the renewed tightening of lockdown restrictions and did not adapt as well to the ongoing pandemic situation as older individuals or men. Thus, these vulnerable groups might need tailored support to prevent a further escalation of symptoms. Surprisingly, the observed long-term increase in depression was much more pronounced in individuals without a history of mental disorders, while the level of depressive symptoms persisted on a high level over the 1-year follow-up period in those with a history of mental disorders.

The present study should be considered in the light of the following limitations: First, in the present study, participants were recruited via convenience sampling methods which may lead to biases in the recruited sample (over- or under-representation of population groups) and, thus, may limit the generalizability of the present findings to the general population of Germany. In fact, as a result of our recruitment method (i.e., convenience sampling methods), in the present sample, older respondents and men as well as individuals with a lower educational level were relatively underrepresented, which might limit the generalizability of the findings, especially to these population subgroups. Thus, the present findings should be validated using representative probability samples. A relatively high number of participants lost to follow-up (45% of respondents participated in at least two assessment waves) which, however, is within the expected attrition rate ranging between 30 and 70% for longitudinal studies [11, 13, 32, 42]. Notwithstanding this, the relatively high attrition rate in the present study should be considered when interpreting the present results with regard to the generalizability of the findings to the general population. Our study exclusively relied on self-report data which might have been subject to memory and recall biases. Please also note that we mainly focused on internalizing symptoms (i.e., depression and anxiety). Thus, additional studies are needed to investigate whether long-term changes during the COVID-19 pandemic were similar for externalizing symptoms (e.g., anger, aggression, alcohol abuse) [6]. Moreover, during all assessment waves, the stringency of lockdown measures was relatively high and comparable across all assessment waves. However, there is evidence that general mental health problems as well as depressive and anxiety symptoms significantly decreased during summer 2020 and 2021, i.e., during easing of lockdown restrictions in Germany and other European countries [10, 13, 22, 23, 35, 36]. For example, data from a longitudinal study in Germany revealed a decrease in depressive and anxiety symptoms from April to June 2020, i.e., during easing of the first lockdown [23]. Thus, it might be that, after an initial reduction in psychopathological symptoms during easing of the lockdown in Germany, symptoms subsequently increased due to the tightening of lockdown restrictions. However, due to the relatively low temporal resolution of the assessment waves, in the present study, we were not able to reveal such potential changes.

## Conclusion

In the present longitudinal observational study, we found no symptom change in the short run but a worsening of depressive symptoms, loneliness and life satisfaction in the long run. Younger individuals were identified as a risk group for overall higher levels of mental health problems and unfavorable trajectories of mental health outcomes. In line with vulnerability-stress models [24], the observed worsening of depressive symptoms may increase the risk for the onset or further deterioration of psychological disorders which may lead to a greater need for psychiatric or psychological treatment. This risk for developing psychopathological symptoms might be further increased in vulnerable groups (e.g., younger individuals) due to the overall higher psychopathological symptoms already present during the initial phase of the COVID-19 pandemic. Therefore, to prevent or mitigate these adverse long-term mental health consequences, interventions or prevention strategies should be implemented, especially in vulnerable populations. Specifically, according to the results of the present study, these interventions should target feelings of social isolation, loneliness and life satisfaction to counteract deterioration or persistence of anxiety and depressive symptoms. For example, based on evidence indicating that higher level of social support and more frequent social contacts were associated with lower depressive symptoms [14, 16, 43], interventions should target at strategies to boost social support and increase the number of social contacts. However, given that we found increases in mental health problems in individuals not identified as at-risk persons in previous studies (e.g., individuals with no history of mental disorders), special attention should also be paid to the long-term trajectories of people who are not supposed to be at higher risk for adverse mental health consequences.
